# Fast and fierce versus slow and smooth: Heterogeneity in immune responses to *Plasmodium* in the controlled human malaria infection model

**DOI:** 10.1111/imr.12811

**Published:** 2019-10-12

**Authors:** Xi Zen Yap, Matthew B. B. McCall, Robert W. Sauerwein

**Affiliations:** ^1^ Department of Medical Microbiology Radboud University Medical Center Nijmegen The Netherlands; ^2^ Radboud Center for Infectious Diseases Radboud University Medical Center Nijmegen The Netherlands

**Keywords:** heterogeneity, IFN‐γ, inhibitory ligands, malaria, T cell, vaccine

## Abstract

Controlled human malaria infection (CHMI) is an established model in clinical malaria research. Upon exposure to *Plasmodium falciparum* parasites, malaria‐naive volunteers differ in dynamics and composition of their immune profiles and subsequent capacity to generate protective immunity. CHMI volunteers are either inflammatory responders who have prominent cellular IFN‐γ production primarily driven by adaptive T cells, or tempered responders who skew toward antibody‐mediated humoral immunity. When exposed to consecutive CHMIs under antimalarial chemoprophylaxis, individuals who can control parasitemia after a single immunization (fast responders) are more likely to be protected against a subsequent challenge infection. Fast responders tend to be inflammatory responders who can rapidly induce long‐lived IFN‐γ^+^ T cell responses. Slow responders or even non‐responders can also be protected, but via a more diverse range of responses that take a longer time to reach full protective efficacy, in part due to their tempered phenotype. The latter group can be identified at baseline before CHMI by higher expression of inhibitory ligands CTLA‐4 and TIM‐3 on CD4^+^ T cells. Delineating heterogeneity in human immune responses to *P. falciparum* will facilitate rational design and strategy towards effective malaria vaccines.

## 1 HETEROGENEITY IN MALARIA

In 2017 there were 219 million reported cases of malaria, posing a substantial global health challenge that disproportionately affects children and pregnant women.^1^ Public health measures and novel drugs have substantially reduced the malaria burden, but the rise of drug‐resistant strains^2^ threatens to undo the positive gains of recent years. A highly effective malaria vaccine would be an invaluable tool in reducing the burden of disease, while only a modestly protective vaccine is currently available.^3^, ^4^

Malaria vaccines frequently demonstrate high efficacy when tested in Phase I/IIa trials in healthy malaria‐naive adult volunteers using controlled human malaria infection (CHMI) models.^5^, ^6^, ^7^, ^8^ Yet when tested under field settings during Phase IIb trials in malaria‐endemic countries, efficacy in such prior‐exposed populations is subpar, particularly in children and infants.^9^ Moreover, vaccine efficacy also varies within both populations, with some immunized individuals displaying protective immunity whilst others remain susceptible.^3^, ^10^, ^11^, ^12^ Individual, qualitative and quantitative heterogeneity in antimalarial immune responses presumably plays a large role. Various underlying factors have been proposed to explain such immunological heterogeneity, both between and within populations, including subject age,^13^, ^14^ genetics,^15^ modulating effects of prior exposure to malaria^16^ and co‐infections.^17^, ^18^ While different underlying factors may exert similar or disparate influences through shared upstream pathways, an individual's immune response represents the aggregate of multiple such factors. Exploring heterogeneity in immune responses to malaria will not only help to shed light on elusive correlates of protection but may also suggest strategies to overcome sub‐optimal vaccine take, eg through improved vaccine design, even if the underlying cause thereof remains to be identified.

Controlled human malaria infection studies have played a major role in clinical vaccine development and our understanding of antimalarial immunity. The standardized protocol, precise measurement of exposure, and reproducible outcomes of CHMI studies provide an ideal setting to study heterogeneity in immune responses to malaria and its potential underlying factors.

## 2 FRIEND OR FOE? A HISTORY OF CONTROLLED HUMAN MALARIA INFECTION

In 1927, psychiatrist Julius Wagner‐Jauregg was awarded the Nobel Prize in Medicine for his pioneering work in curing neurosyphilis^19^ using the malaria‐causing parasite *Plasmodium*. The concept in itself was not radical: famous physicians of antiquity, including Hippocrates and Galen, had noted 2000 years ago that patients with psychosis improved after being afflicted with “intermittent fever”, the cyclical fever characteristic of malaria.^20^ Yet Wagner‐Jauregg's work inadvertently provided the basis for a model that exists today, and is among our most powerful tools to get to grips with the same *Plasmodium* parasite that Wagner‐Jauregg first employed to save syphilis patients.

After Wagner‐Jauregg published his findings, the medical field was galvanized. The gruesome and often fatal symptoms of syphilis were a potent incentive to adopt the new, seemingly safe technology of malariatherapy, where malaria was easily cured by a dose of quinine at 7 to 12 days postinfection, during the peak of fever. Originally, Wagner‐Jauregg inoculated his neurosyphilis patients by subcutaneous injection with blood of malaria‐infected soldiers returning from World War I.^19^ Later he would inject the infected blood of treated patients into untreated patients, claiming to have maintained one strain through two hundred passages through humans.^21^ Physicians in the USA and Britain expanded the technology by transferring malaria through the bites of infected female *Anopheles* mosquitoes, the parasite's natural vector.

By the 1940s, malariatherapy for syphilis patients had been succeeded by antibiotics. But malaria was still being deliberately induced, now among the inmates of US state penitentiaries, as part of a military program researching new treatments and prophylaxis for soldiers deployed to malaria‐endemic areas.^22^ By the end of 1946 approximately 500 prisoners had been infected with the supposedly benign species *Plasmodium vivax*.^23^ In addition to facilitating testing of experimental antimalarial drugs, the information derived from the prison studies has since been used to model *P. vivax* kinetics in vivo,^24^ providing valuable insights into a species whose unique biology has hindered research efforts.^25^, ^26^ In 1986, the first controlled human infection of *Plasmodium falciparum* in healthy volunteers was conducted at the Walter Reed Army Institute of Research in the USA, where six volunteers were infected via the bites of laboratory‐reared infectious mosquitoes.^27^ The very next year, this method was used to test the efficacy of a recombinant peptide vaccine candidate against *P. falciparum* malaria in experimentally infected volunteers.^28^

### 2.1 Controlled human malaria infection in modern times

**Figure 1:**
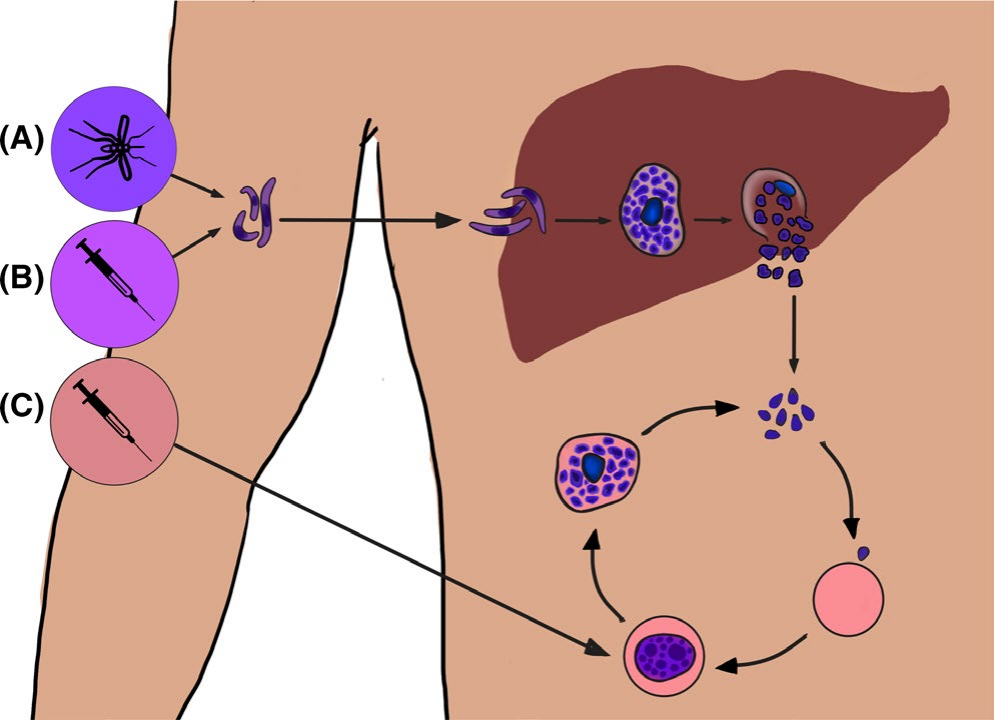
Controlled human malaria infection. The *Plasmodium* life cycle begins in humans when sporozoites are injected into the skin. They make their way to the liver and invade hepatocytes, where they mature into an intra‐erythrocytic schizont. Schizont rupture releases invasive stages known as merozoites into the bloodstream, where they invade host erythrocytes, mature into blood‐stage schizonts, and lyse to resume the cycle afresh. Taking advantage of the complex life cycle, controlled human malaria infection can be induced through the administration of sporozoites (A and B) or infected red blood cells (C). Infected mosquito bites (A) deliver sporozoites into the skin, while needle‐and‐syringe administration (B) delivers cryopreserved sporozoites into the vasculature. Sporozoites travel from the administration site to the liver, where they replicate and eventually emerge into the bloodstream. Alternately, (C) infected red blood cells can be directly administered into the blood stream, bypassing the liver and directly commencing blood‐stage replication

From 1986 to 2019, 84 CHMI trials have been conducted, mostly to test novel vaccines or drugs. *Plasmodium* parasites have a complex life cycle spanning sexual replication in mosquitoes and asexual replication in humans (Figure 1). It is the blood‐stage asexual multiplication cycle that is responsible for pathology and clinical symptoms. Lysis of these parasitized red blood cells (pRBC) releases a range of inflammatory products^29^ with immunomodulatory effects.

**Figure 2:**
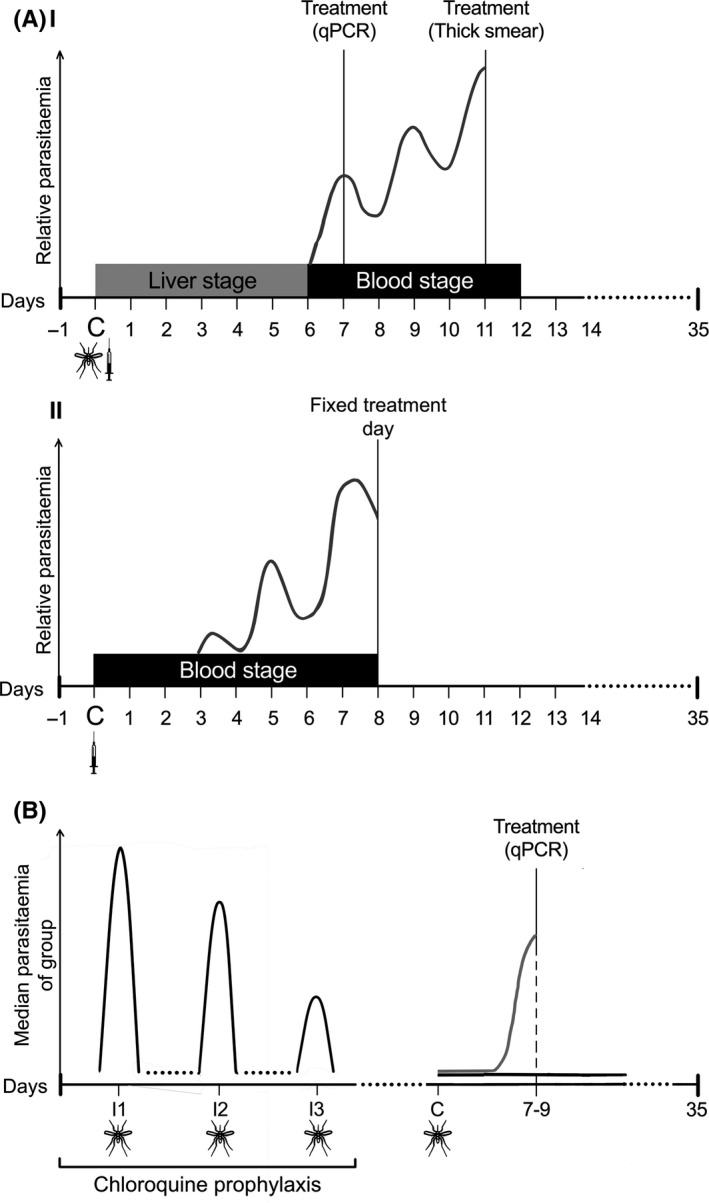
Dynamics of parasitemia in different controlled human malaria infection models. Approximate parasite density is shown on the *y‐*axis. A, (i) Controlled human malaria infection through administration of sporozoites results in emergence of blood‐stage parasites from the liver at approximately days 6‐7 postchallenge. Treatment is administered once parasitemia reaches a certain threshold as measured using qPCR or light microscopy. (ii) Induced blood‐stage malaria through intravenous administration of blood‐stage parasites results in a steady increase in circulating parasitemia until a set day of treatment, usually 7‐8 d post‐challenge. In both cases participants are monitored until the resolution of infection and samples are usually collected at day 35 post‐challenge to examine the changes in host immunity brought about by CHMI. B, Chemoprophylaxis with *Plasmodium* sporozoite immunization is carried out by immunizing volunteers through the bites of *Plasmodium‐*infected mosquitoes (black) repeatedly under cover of quinoline chemoprophylaxis. A control group is exposed to a similar regimen with the bites of uninfected mosquitoes (data not shown). Black lines illustrate the kinetics of immunized volunteers: the median parasitemia of the group decreases with successive immunizations, and during rechallenge after cessation of chloroquine cover, immunized volunteers either have delayed time to parasitemia or are completely protected. In contrast, the control group (gray line) develops parasitemia as in normal controlled human malaria infection

Parasite administration for CHMI can be achieved in a variety of ways (Figure 1): (a) administration of sporozoites by a predetermined number of laboratory‐reared infected mosquito bites^30^, ^31^, ^32^, ^33^; (b) needle‐and‐syringe administration of a fixed number of sporozoites via direct venous injection (DVI)^34^, ^35^, ^36^; (c) DVI of a fixed number of pRBC.^37^, ^38^, ^39^, ^40^ Mosquito bites most closely recapitulate the natural course of infection. DVI of sporozoites bypasses the skin and subsequently tissue‐resident immunity and/or tolerance.^30^ DVI of pRBC bypasses liver and skin and directly induces blood‐stage infection. In sporozoite CHMIs, volunteers are given curative treatment once parasitemia is detected using quantitiative polymerase chain reaction (qPCR)^41^ or light microscopy^42^ (Figure 2Ai) whereas in pRBC CHMI, treatment is administered on a fixed day post‐challenge (Figure 2Aii). In these studies it is normal for 100% of previously malaria‐naive volunteers to develop patent parasitemia, meaning that high statistical power can be achieved with a small sample size.^43^, ^44^, ^45^

Emerging parasitemia does cause mild to moderate malaria symptoms including malaise and headache, and occasionally severe symptoms in some volunteers. The insight gained from these small‐scale clinical trials, however, acceptably counterbalances the potential risks to volunteers.^43^, ^46^ Therefore, their safety and reproducibility have established CHMI as a scientifically robust, ethically accepted and cost‐effective tool in various fields of clinical malaria research.

### 2.2 CHMIs as a model to study immunity

Besides clinical evaluation of candidate vaccines and drugs, CHMI also represents a prime model to dissect fundamental immunological questions. These include mechanisms of protective immunity as well as the search for a persistently elusive immunological correlate of protection. Immune responses can be induced by and potentially act upon both sporozoites and intra‐hepatic parasites (together termed pre‐erythrocytic immunity) and merozoites and intra‐erythrocytic parasites (blood‐stage immunity). Indeed, despite the relatively small window of exposure, even a single CHMI is sufficient to induce antimalarial responses against both these stages of the parasite's lifecycle.

Since CHMIs are conducted with *Plasmodium* strains originally isolated from patients with malaria, these models are closely representative of natural infections and have direct clinical and immunological relevance. The main strength of CHMI models lies in their defined timing and inoculum dose, allowing associations to be defined between exposure, immune responses and ultimately protection, both at the individual level and between experimental groups. In field studies, in contrast, it is difficult or impossible to determine how frequently or recently a participant has been previously exposed to malaria.

For logistical reasons, CHMIs have historically been performed primarily in malaria‐naive (largely Caucasian) populations in Europe, the USA and Australia, but transfer of technology has recently allowed such studies to also be conducted in malaria‐endemic countries.^47^, ^48^ Performing CHMI in naive populations eliminates the potential confounder of prior malaria exposure. Alternately, CHMI in endemic populations with prescreening to measure, and if desired stratify by, levels of prior exposure is a novel and controlled way to examine efficacy and longevity of naturally acquired immunity.^47^, ^48^ There is also potential to assess how different levels of pre‐existing immunity affect pathology, which will become increasingly relevant in areas where malaria transmission is declining.^49^

Here we have reviewed data on immunology generated by (single) CHMIs and outline how heterogeneity manifests across a range of immune responses. Heterogeneity is also discernible in the development of sterile immunity in a repeated‐exposure model of CHMI, particularly the dynamics thereof. Based on these insights, we propose an underlying immunological mechanism which predicts heterogeneity in response to malaria in CHMI volunteers, and hypothesize on how this could answer fundamental questions about individual differences in vaccine responsiveness. Unless otherwise noted, this insight derives from CHMIs involving *P. falciparum* infection in malaria‐naive volunteers, as this represents the overwhelming majority of CHMI studies conducted until now.

## 3 IMMUNE RESPONSES IN SINGLE CHMI

### 3.1 Kinetics of circulating immune cells

A significant reduction in circulating immune cells at or shortly after the day of treatment is a common feature post‐CHMI.^50^, ^51^, ^52^, ^53^, ^54^ This is likely due to systemic inflammation and complement activity caused by circulating parasites.^55^ The greatest reductions are observed in lymphocytes and respective subpopulations including natural killer (NK) cells, γδ T cells, αβ T cells, innate lymphoid cells subset 1 (ILC1), mucosal‐associated invariant T cells (MAITs), and invariant natural killer cells (iNKT) coincident with the emergence of merozoites from the liver.^50^, ^51^, ^52^, ^53^, ^54^ Post‐curative treatment, at the final day of study (commonly day 28 or 35 post‐challenge), frequencies and numbers of these subsets mostly return to normal.^50^, ^54^ Numbers of γδ T cells can remain elevated for months to years after a single CHMI.^56^ MAIT cell populations are similarly maintained up to six months post‐CHMI but do not have enhanced functional activity or activation marker expression^52^; their role in antimalarial responses remains unclear. Crucially, these lymphocyte subpopulations all produce IFN‐γ, a major player in heterogeneity of the antimalarial immune response. Understanding their kinetics during acute infection is insightful for assessing how quickly these responses are initiated.

The roles of other leukocytes in heterogeneity are less clearly defined but their kinetics are largely similar to those of lymphocytes. As parasites enter the peripheral blood at seven days postinfection (C + 7; Figure 2Ai), monocytes and neutrophils generally decrease in the periphery,^50^, ^54^, ^57^ though monocytes can also remain stable.^53^ The relatively low proportion of monocytes in the circulation makes it difficult to identify statistically significant changes. A reduction in neutrophil counts and degranulation activity during merozoite emergence is also observed in *P. vivax* CHMI.^57^ It is likely that loss of cells from the circulation is partially due to sequestration, as evidenced by upregulation of cytokines that promote cellular migration, particularly as a large proportion of functional responses are indicated to take place in the liver^58^ and lymphoid tissues. Despite individual differences in cell proportions at baseline, the timing and pattern of changes in peripheral immune cell numbers and proportions appears remarkably consistent across CHMI volunteers. Heterogeneity is reflected more by the phenotype and function of these cells.

### 3.2 Cytokines and inflammatory factors

The one cytokine that is upregulated in a majority of CHMI volunteers is IFN‐γ. Concentrations of IFN‐γ peak on the day of treatment or soon after, correlating with parasitemia.^59^, ^60^, ^61^, ^62^, ^63^, ^64^, ^65^, ^66^ IFN‐γ also enhances expression of CXCL9^65^, ^66^, ^67^ and release of soluble granzyme A and B,^59^ suggesting enhancement of cytotoxic cell activity. However, high proportions of IFN‐γ‐producing malaria‐specific T cells are associated with lower levels of protective antibodies.^68^, ^69^, ^70^ This may be due to IFN‐γ driving production of B cell regulatory cytokine BAFF,^63^ or suppressing the antibody‐promoting T follicular helper cell 2 (Tfh2) lineage (reviewed in Ref. 71).

A wide variety of other inflammatory cytokines are produced during CHMI including IL‐12p40,^59^ IL‐12p70,^60^ IL‐2,^62^ IL‐8,^53^, ^59^, ^60^ IL‐1b,^60^ IL‐6 and TNF‐α.^60^, ^61^, ^72^ Interestingly, IFN‐γ, IL‐8, IL‐1b, IL‐6 and TNF‐α are all regulated by the NFκB signaling pathway. IL‐12 is also a key differentiation factor for type 1 IFN‐γ‐producing lymphocytes, suggesting a central role for IFN‐γ signaling or its underlying pathway. The abundance of inflammatory cytokines also induces production of IL‐10, which is elevated from the day of treatment (DT) onwards.^59^, ^60^

There is substantial variation between volunteers in the quantity and type of cytokines produced in the initial response to parasitemia. Peripheral cytokine responses are highly complex, but this first‐wave immune response may nonetheless determine heterogeneity between downstream cellular responses of malaria‐exposed volunteers. The patterns of cytokine responses after CHMI can be characterized by either an initial IFN‐γ response followed by IL‐10, sometimes with intermediate IL‐12p70; or by expression of TGF‐β without further downstream inflammatory responses.^60^ Individuals who express IFN‐γ phenotypes, hereon referred to as “inflammatory responders”, are associated with more inflammatory symptoms and better parasite control. In contrast the TGF‐β phenotype, which is characteristic of “tempered responders”, results in fewer symptoms but higher parasitemia.^60^, ^64^ CD14^+^ monocytes are the main producers of TGF‐β in this context, along with a large population of indeterminate cells,^64^ while Foxp3^+^ cells also stimulate TGF‐β production.^64^ In Section 3.5 we hypothesize on how these phenotypes may impact the relationship between IFN‐γ and antibody responses.

### 3.3 Innate cell responses to a single CHMI

Antigen presenting cells (APCs), namely monocytes and dendritic cells (DCs), are required for induction of adaptive immunity but appear to be dysregulated during CHMI. Phagocytosis and antigen uptake is downregulated,^73^, ^74^, ^75^ antigen presentation is reduced,^57^, ^65^, ^73^, ^75^, ^76^ apoptosis increases,^73^, ^75^ there is increased induction of IL‐10‐producing regulatory T cells (Treg),^77^ and they upregulate inhibitory ligand LAG‐3, a negative regulator of T cell proliferation.^57^ Yet they also express co‐stimulatory markers, particularly during peak parasitemia and post‐treatment,^75^, ^76^, ^78^, ^79^ suggesting that APCs are activated but may be polarized to initiate regulatory responses. IFN‐γ also upregulates the immunosuppressive IDO pathway in DCs,^74^ pointing to a role for IFN‐γ in blocking APCs from initiating humoral immunity to malaria. Thus, APCs may contribute to heterogeneity through their key roles in modulating both the humoral and cellular arms of adaptive immunity.

### 3.4 T cell responses after a single CHMI

T cells, NK cells, and γδ T cells are the main producers of IFN‐γ during CHMI, and the proportion of IFN‐γ^+^ cells in each subset increases in response to parasitemia.^56^, ^80^ Of these, T cells constitute the majority of pRBC‐specific IFN‐γ^+^ producing cells,^56^, ^80^ and pRBC‐specific IFN‐γ^+^ T cells remain stable even four months postchallenge while the pRBC‐targeting IFN‐γ^+^ NK cell population wanes relatively quickly. Selective subset depletions show that CD3^+^ cells, especially T cells, are the master regulators of IFN‐γ memory responses, while antimalarial NK cell responses require T cell help.^80^ Some loss of IFN‐γ^+^ cells in CD3^+^‐depleted cultures may be due to loss of γδ T cells, which constitute a substantial and long‐lived population of IFN‐γ^+^ cells in CHMI.^56^ CHMI‐induced T cells therefore appear to have substantial influence over the IFN‐γ^+^ memory response, affecting longevity and responsiveness of other cell populations. Heterogeneity in the T cell population may cause knock‐on effects on other cell subsets. Small delays in individual capacity to induce malaria‐specific IFN‐γ^+^ T cells may translate into substantial cumulative deficits in antimalarial immunity and more rapid parasite multiplication.

Indeed, marked heterogeneity exists among CHMI subjects with regards to their T cells’ ability to develop this protective response in CHMI. T cells of inflammatory responders are characterized by the protective IFN‐γ‐producing phenotype.^56^, ^64^, ^68^, ^69^, ^70^, ^81^, ^82^ A proportion of these IFN‐γ^+^ cells co‐express one or multiple IL‐2^+^,^69^, ^82^ IL‐4^+^,^68^, ^69^ and TNF‐α^+^,^69^, ^82^ and the majority have increased proliferative potential when re‐encountering malaria in vitro.^69^ A potential subpopulation that may contribute to the protective cellular response are a novel population of CD4^+^CD38^+^ αβ T cells which have low expression of cytokines including IFN‐γ, but are nonetheless associated with control of blood‐stage parasitemia through their increased cytotoxic activity.^83^ They have much higher expression of CD69, granzyme B, and perforin at baseline and at peak parasitemia than CD38^−^ counterparts.^83^ Higher numbers of CD38^+^CD4^+^ cells are not associated with higher titers of blood‐stage antibodies,^84^ suggesting that parasite control by these cells is purely cytolytic.

In contrast, tempered responders generate increased and sustained numbers of Tregs and have increased concentrations of circulating TGF‐β. This is associated with increased parasite growth rates, likely due to suppression of protective inflammatory responses including IFN‐γ.^64^, ^69^ These Tregs exert suppressive bystander effects upon unrelated T cell responses, including the development of central memory T cells.^69^

Successful induction of CD4^+^ T cells therefore seems paramount for successful immunity.^84^ It warrants further examination as to whether individuals are predisposed to generate a particular T cell phenotype, and particularly whether these phenotypes are mutually exclusive. IFN‐γ^+^ cell differentiation is likely suppressed by Treg in individuals predisposed to regulatory responses, but the role of the CD38^+^ subset remains unclear.

### 3.5 Humoral responses after a single CHMI

Antibody responses are readily generated in CHMI despite a relatively short duration of exposure. The presence of class‐switched antibodies, IgG and IgA, up to five months postchallenge denotes how even a single CHMI can result in generation and maintenance of a humoral memory population.^68^, ^84^, ^85^, ^86^ Antibodies are generated against a wide range of blood‐ and pre‐erythrocytic stage targets (MSP‐1, MSP‐3, AMA‐1, PfEMP1, GLURP, CSP, EXP‐1, LSA‐1, whole sporozoite lysate, whole pRBC lysate^49^, ^66^, ^68^, ^70^, ^81^, ^84^, ^85^, ^86^, ^87^, ^88^, ^89^, ^90^, ^91^). CHMI‐derived antibodies display functional activity in vitro, such as blocking hepatocyte invasion^85^ and enhancing neutrophil release of reactive oxygen species.^49^ Induction of IgG1 and IgG3 subclasses^68^, ^84^ suggests that CHMI‐induced antibodies may also protect against malaria by opsonizing parasites^92^ and fixing complement.^93^

However, not all individuals produce significant quantities of functional antibodies in response to CHMI. Some individuals are predisposed to develop cellular responses, while others favor humoral responses. A negative correlation has been observed between numbers of pRBC‐specific IFN‐γ^+^ T cells and titers of anti‐MSP‐1_19_ IgG.^68^ In a CHMI of Tanzanian volunteers, individuals with comparable histories of exposure diverged during challenge into inflammatory IFN‐γ^+^‐type responders or humoral responders.^70^ It is unclear whether humoral responses are generated to compensate for the lack of cellular responses or vice versa; the necessity of T cell help for memory B cell (MBC) differentiation and class‐switching suggests that B cell responses are secondary. These two phenotypes correlate broadly with the inflammatory and tempered responder phenotypes, with the latter being antibody producers.

### 3.6 Immune heterogeneity in single CHMI: inflammatory and tempered responders

As touched upon in the sections above, subjects undergoing CHMI appear to fall into two broad groups, encompassing a suite of respective responses across multiple arms of the immune system:

1. *“Inflammatory responders”* are characterized by high expression of IFN‐γ,^60^ primarily from induction of parasite‐specific IFN‐γ^+^ T cells.^56^, ^64^, ^68^, ^69^, ^70^, ^81^, ^82^ These cells control parasitemia^69^ by the production of inflammatory cytokines^56^, ^68^, ^69^ and granzyme‐mediated lysis of infected cells.^59^ They also help to maintain other populations of IFN‐γ^+^ lymphocytes such as NK cells.^80^ This response is self‐propagating: IFN‐γ induces production of CXCL9 and CXCL10,^65^, ^66^, ^67^ which bind CXCR3, promoting T cell polarization towards the IFN‐γ‐producing Th1 lineage. However, the latter may lead to loss of humoral immunity due to sustained proliferation of Th1 cells at the expense of the antibody‐promoting Th2 lineage.
2. “*Tempered responders”,* who express TGF‐β upon initial parasite exposure, induce Tregs and IL‐10‐producing cells.^60^, ^64^, ^69^, ^77^ This results in suppression of IFN‐γ^+^ T cell responses, abrogating the disruptive effects of prolonged IFN‐γ signaling on humoral immunity and permitting development of protective antibody responses. IFN‐α production from cDCs and NK cells may be another regulatory mechanism in this group, as it induces Tregs which subsequently suppress IFN‐γ responses through the production of IL‐10.^77^

These distinct responses to CHMI may suggest that induction of protective antibodies to malaria may actually represent a backup response after failure to induce protective IFN‐γ^+^ T cells. The correlation between antibody and MBC responses with length of blood‐stage exposure and peak parasitemia^66^, ^68^, ^90^ lends support to the notion that antibodies represent at least as much a correlate of exposure, as of protection. Protection against naturally acquired malaria is held to be antibody‐mediated, whereas T cells are often reported to be less functional, displaying an exhausted phenotype characterized by high expression of inhibitory ligands.^94^, ^95^, ^96^ In endemic areas, chronic parasitemia induces persistent inflammation which drives tolerization and downregulation of IFN‐γ^+^ T cells. Humoral immunity can then be generated, either due to absence of suppressive IFN‐γ or due to high levels of exposure, and results in protection after multiple exposures. The division in the Tanzanian cohort between IFN‐γ‐producers and antibody producers, who had similar histories of exposure, is evidence for these phenotypes in a field setting.^70^

Single CHMIs are nevertheless inadequate to assess how these responder phenotypes affect long‐term development of immunity. Multiple CHMIs rather than single infections are required to examine the kinetics and especially effectiveness of IFN‐γ‐driven responses for induction of long‐lasting protective immunity. Tailoring the CHMI design has thus allowed us to delve deeper into the development, kinetics, and longevity of sterile immunity.

## 4 HETEROGENEITY IN CONTROLLED SPOROZOITE IMMUNIZATION UNDER CHEMOPROPHYLAXIS

Complete sterile protection can be attained in the CHMI model when volunteers under chloroquine^61^, ^67^, ^97^ or mefloquine^98^ chemoprophylaxis are exposed three times to 5‐15 infected mosquito bites^67^, ^97^, ^98^, ^99^, ^100^ or injections of cryopreserved sporozoites^101^, ^102^, ^103^ at monthly intervals (Figure 2B). This immunization regimen of chemoprophylaxis with sporozoite (CPS) induces sterile protection to a challenge with homologous parasite strains for up to 28 months.^61^, ^97^, ^101^

In CPS, qPCR measurement of parasitemia after each immunization provides parasitological correlates for immunological observations, allowing us to identify how quickly some volunteers become sterilely immune. Assessing with this model the factors that distinguish inflammatory and tempered responders in CHMIs can add an extra dimension to the hierarchy of T and B cell responses outlined previously. Are the volunteers who have more effective T cell responses more able to rapidly develop sterile immunity? It is possible that IFN‐γ^+^ CD4^+^ T cell responses represent the primary mechanism of protection in CPS. Sampling across the course of multiple immunizing infections permits a closer examination of the kinetics, efficacy, and maintenance of antimalarial responses in relation to (precisely quantified) exposure.

### 4.1 Cellular immunity induced by CPS immunization

CPS immunization leads to expansion of malaria‐specific, IFN‐γ^+^ T cells that recognize both sporozoites and pRBCs.^56^, ^61^, ^97^, ^104^ This cell population expands during both immunization and challenge^56^, ^105^ and is maintained in CPS vaccinees up to 28 months postimmunization.^56^, ^61^, ^97^ The majority possess an effector memory phenotype (CD62L^−^CD45RO^+^), though a small population of central memory cells is also induced.^61^, ^97^, ^105^ High levels of CD4^+^ T cells positive for the degranulation marker CD107a^98^, ^104^ and CD8^+^ T cells positive for granzyme B associate strongly with protection,^104^ indicating that direct lysis of infected cells is important for parasite control. CD107a^+^ CD4^+^ T cells also have significantly higher granzyme B and IFN‐g expression than CD107a^−^ cells, both at baseline and postimmunization.^104^ IFN‐g^+^CD107a^−^ cells are also likely to play a protective role not dependent on cytotoxic activity.

Υδ T cells are known to be major producers of IFN‐γ in response to malaria. Even at baseline they constitute a large fraction of the malaria‐recognizing IFN‐γ^+^ population, and are an important component of the IFN‐γ memory response, continuing to recognize malaria antigens up to six months postchallenge.^56^ Their proportions, numbers, and expression of granzyme B increase with each successive immunization,^56^, ^105^ but in individuals who are not sterilely protected after one immunization, γδ T proliferation specifically increases in response to parasitemia after the second immunization.^105^ Υδ T cells may therefore have an important protective role in individuals who are unable to quickly generate sterile protection.

Interestingly, the majority of malaria‐specific IFN‐γ^+^ T cells and γδ T cells, and protective CD107a^+^ CD4^+^ T cells, are induced by the first immunization.^104^, ^105^ In subsequent immunizations the population remains stable in protected volunteers or declines in unprotected volunteers. Moreover, the size of the CD107a^+^ CD4^+^ population induced during that first immunization is smaller in unprotected volunteers.^104^ It therefore appears that it is possible to identify volunteers who will be protected from as early as the first immunization, and that the protective response is similar to the inflammatory phenotype earlier described in CHMI. Furthermore, individuals who do not develop this T cell‐mediated protective response early on will not do so even in subsequent immunizations.

### 4.2 Humoral immunity induced by CPS immunization

CPS immunization induces a heterogeneous range of antibody responses to pre‐erythrocytic and blood‐stage targets (CSP, LSA‐1, TRAP, LISP2, MSP‐1, AMA‐1, PfEMP1, asexual lysate^67^, ^97^, ^98^, ^100^, ^106^, ^107^, ^108^, ^109^) and a range of proteins of unknown function.^106^ In contrast, however, antibodies against the dominantly expressed CSP are by far the most commonly reported target of CPS‐generated antibodies.^67^, ^97^, ^98^, ^100^, ^106^, ^107^, ^109^ Moreover these antibodies show a variety of functions in vitro, being able to inhibit sporozoite traversal of hepatocytes,^109^, ^110^ facilitate complement‐mediated sporozoite lysis,^111^ and block sporozoite development in human hepatocytes in vitro^99^, ^108^ and in mice with humanized livers.^110^

Specific antibody titers decrease after immunization, while memory B cells remain stable over the same period.^100^ This may be related to the relative increase of CPS‐induced IgM^+^ rather than IgG^+^ plasmablasts with each immunization.^109^ While repeated immunizations increase the titer and efficacy of humoral responses, a subpopulation of volunteers indeed generate specific memory B cells after a single CPS immunization and are able to maintain this population until challenge.^100^, ^109^

Interestingly and somewhat counter‐intuitively, antibody specificities against a broader range of targets is associated with lower protective efficacy, as shown in microarray studies.^106^, ^107^ This may be due to a relatively slower development of cellular immunity over the course of CPS immunization, resulting in a higher cumulative parasite load of blood‐stages in particular.^100^ Thus the high antigenic load in the blood‐stage results in antibody generation against more targets, reflecting upstream failure to induce protection against pre‐erythrocytic stages. Parallels can be drawn between these individuals who fail to generate protective cellular responses and the tempered responders observed in CHMI.

### 4.3 Heterogeneity in immune response after CPS immunization: fast and slow responders

**Figure 3:**
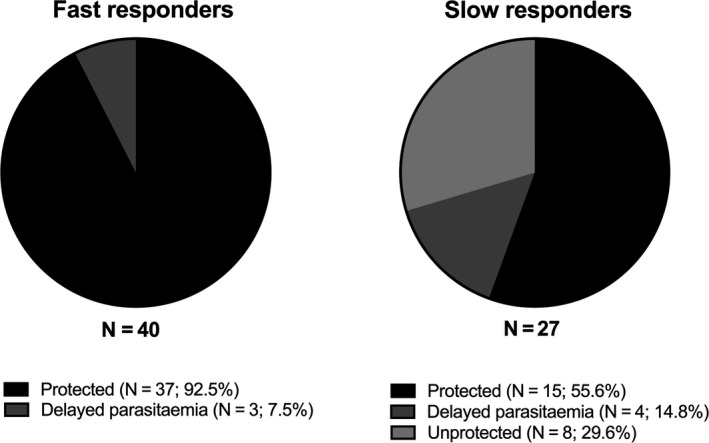
Fast and slow responders in chemoprophylaxis with sporozoite (CPS) immunization. Six CPS immunization studies comprising 67 homologously challenged volunteers were studied for the presence of fast and slow responders. Forty‐seven volunteers (60%) were PCR‐negative for the presence of parasites at the second immunization and were classified as fast responders. Of these, 37 (92.5%) were fully protected upon challenge. Only 15 slow responders (55%) were fully protected upon challenge

The vast majority (52/67; 78%) of CPS volunteers will be protected against homologous challenge after completing three immunizations. In a proportion of volunteers (40/67; 59.7%), one immunization is sufficient to induce full sterilizing protection. These individuals are referred to as “fast responders”, while their “slow responder” counterparts require two or more immunizations to become immune. RNAseq data show that the greatest magnitude of transcriptomic difference is found after the first immunization for protected volunteers, but only after the third immunization for non‐protected volunteers, suggesting that slow responders are less likely to be protected.^112^ This pattern is reflected by the kinetics of protective CD107a^+^ CD4^+^ T cells, which in the protected group are elevated by the first immunization,^104^ and by clinical data. In six CPS studies^67^, ^99^, ^104^, ^105^ only 15/27 (55.5%) of slow responders were fully protected against homologous challenge, as opposed to 37/40 (92.5%) of fast responders (Figure 3). Moreover, the fraction of non‐protected fast responders presents with significantly lower parasitemia after the challenge infection (Fast: 122.2 parasites/mL, 95% CI −5.9‐250.2; Slow: 3734 parasites/mL, 95% CI 848.4‐6619; *P* < .0001). Such differences are already apparent during the first immunization (Fast: 1718 parasites/mL, 95% CI 892.4‐2634; Slow: 2839 parasites/mL, 95% CI 1669‐4009; *P* = .0796).

The limited efficacy of antibodies in long‐lived protection after CPS and the correlation between parasite exposure and humoral response efficacy suggest that protective immunity operates differently in fast and slow responders. Fast responders are usually inflammatory responders who induce parasite‐specific IFN‐γ ^+^ and CD107a^+^ T cells, which are the most potent effectors of sterile immunity. They control parasitemia presumably through both direct lytic effects and systemic effects mediated by production of cytokines including TNF‐α and IFN‐γ. Slow responders are usually tempered responders who do not induce sufficient pre‐erythrocytic immunity through IFN‐γ^+^ T cells or other mechanisms, but instead allow humoral immunity to come into play, particularly against blood‐stages. This broadly explains the paradoxical relationship between blood‐stage antibodies and susceptibility. More research will be required to understand the involvement of antibody responses in fast responders. It remains to be clarified to what extent slow responders generate blood‐stage humoral immunity more readily because they fail to induce IFN‐γ^+^ which would otherwise actively *suppress* antibody development, or if the high antigenic load in the blood‐stage that is the primary factor driving antibody production, or a mixture of the two.

It therefore seems that, while there are many roads towards protection, individuals who quickly and efficiently acquire CD4^+^ T cell immunity are most likely to be protected. What is the distinction between CPS vaccinees who rapidly develop T cell immunity and those who fail to do so? While IFN‐γ expression is consistently shown to be key for protection, it is a proximate marker of the protective process rather than its origin. Understanding the underlying processes that differentiate the immunology of a “fast responder” from that of a “slow responder” will enable us to understand which pathway(s) must be targeted to induce sterile protection. As such, is it possible to identify a phenotype that predicts how this web of immune responses will play out within a single individual, and therefore predict immunization success?

## 5 T CELL INHIBITORY LIGANDS AS BIOMARKERS FOR PROTECTIVE DYNAMICS IN CPS IMMUNIZATION

Could the responder phenotypes observed in CPS be explained by the expression of T cell inhibitory ligands on CD4^+^ T cells prior to the first immunization? In the steady‐state, inhibitory ligands are responsible for preventing immunopathology by controlling T cell activation. High inhibitory ligand expression is a hallmark of many cancers.^113^, ^114^, ^115^ In infectious disease, they are most frequently studied as markers of T cell exhaustion after viral infection.^116^, ^117^, ^118^, ^119^, ^120^ Three of the most commonly studied inhibitory ligands are programmed cell death 1 (PD‐1), cytotoxic T‐lymphocyte associated protein (CTLA‐4), T cell immunoglobulin and mucin‐domain containing 3 (TIM‐3).

PD‐1 is one of the most well‐known inhibitory receptors and it, and its natural ligands PD‐L1 and PD‐L2, are highly expressed on activated T cells (reviewed in Ref. 121). The PD‐1 pathway downregulates T cell proliferation, cytokine production, and cytolytic function, and induces apoptosis, and is also involved in the generation of induced Tregs.^121^

CTLA‐4 is highly expressed on Tregs. It is a close homologue of CD28,^122^ but directly competes for CD28’s ligands, the co‐stimulatory markers CD80 and CD86, with higher affinity and avidity.^123^ CTLA‐4 is a potent immunoregulatory ligand, as seen in CTLA‐4 KO mice, which die of massive autoimmune disease driven by lymphocyte overproliferation.^124^, ^125^

TIM‐3 binds to soluble ligands such as the C‐type lectin galectin‐9 and the chromatin high‐mobility group box 1 (HMGB1), as well as cell‐bound ligands like Ceacam‐1 and phosphatidyl serine from apoptotic cells. It is expressed on NK cells, dysfunctional CD8^+^ T cells, and CD4^+^ T cells, especially Th1. It is primarily associated with regulation of IFN‐γ driven inflammation and is an inducer of apoptosis, as well as a regulator of Treg function within inflamed tissues.^113^, ^126^

Studies in several murine malaria models show that inhibitory ligands are frequently associated with increased pathology and higher parasitemia.^127^ PD‐1 signaling reduces CD8^+^ T responsiveness and increases apoptosis.^127^ Combined PD‐1 and LAG‐3 blockade may decrease morbidity by reducing suppression of Tfh.^128^

Naturally acquired malaria in children induces upregulation of CTLA‐4, PD‐1, LAG‐3 and TIM‐3, which are all associated with downregulation of protective immune responses and increased morbidity.^94^, ^95^, ^128^, ^129^, ^130^, ^131^ The mechanism of suppression is partially attributable to Tregs, which typically have high expression of CTLA‐4.^94^

### 5.1 Inhibitory ligand expression and kinetics in fast and slow CPS responders

**Figure 4:**
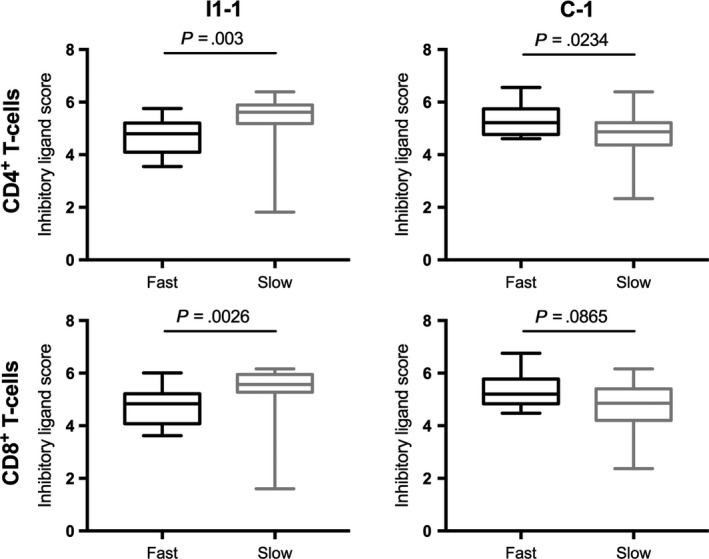
Inhibitory ligand expression at baseline differs significantly between CD4^+^ T cells of fast and slow responders. Inhibitory profiles were generated for each individual by combining *z‐*scores of CTLA‐4, TIM‐3, and PD‐1 at each time point. Inhibitory profiles of slow responders CD4^+^ cells were significantly higher prior to the first immunization (I1‐1). In contrast, inhibitory profiles of slow responders’ CD4^+^ and CD8^+^ T cells were significantly lower by the end of immunization (C‐1), while fast responders’ inhibitory profiles increased. Mann‐Whitney U test was used to calculate significance

In 32 volunteers in two CPS studies, expression of CTLA‐4 is significantly higher on slow responders’ NK cells, γδ T cells, and CD4^+^ and CD8^+^ T cells (unpublished data). Expression of TIM‐3 is significantly higher on γδ, CD4^+^, and CD8^+^ T cells when measured *prior to* first immunization. When all three inhibitory ligand values are combined into a composite *z*‐score for each individual, slow responders’ CD4^+^ T cells express significantly higher inhibitory ligand scores prior to immunization (I1‐1; Figure 4). Thus, higher expression of CTLA‐4 and TIM‐3 are predictive of an individual's ability to generate effective sterile protection in CPS.

This phenotype was shown to predict protection at multiple time points. As early as the first immunization, slow responders show higher parasitemia. During challenge, slow responders also show higher peak parasitemia and shorter time to treatment. Specific in vitro pRBC stimulation of PBMCs collected prior to immunization showed higher upregulation of inhibitory ligands in slow responders, demonstrating that upon encountering malaria they are predisposed to respond to malaria in a regulatory fashion. The combined data indicate that increased inhibitory ligand expression instantly delays generation of cellular and functional protective immune responses.

Interestingly, αβ T cells of fast responders express significantly higher inhibitory scores after the third immunization than their slow responder counterparts, particularly CTLA‐4 and TIM‐3. Therefore, it seems that fast responders acquire effective responses early, but subsequent immunizations induce tolerization that prevents responses increasing further to excessive levels. Conversely, in slow responders, every subsequent immunization stimulates a relatively slow increase of functional responses. This may be so slow that the required threshold of immune responses for protection may not be reached in some volunteers. It also illustrates that to some extent these phenotypes are mutable.

CTLA‐4 and TIM‐3 are powerful negative regulators of T cell function with varied mechanisms of action. It is imperative to understand the immunological pathways through which high CTLA‐4 and TIM‐3 expression is able to delay the development of IFN‐γ^+^ T cell responses to malaria.

### 5.2 Downstream signaling of inhibitory ligands in the fast/slow responder phenotypes

Systems immunology approaches demonstrate that CPS‐protected volunteers preferentially activate NFκB.^112^ NFκB signaling is required for the production of malaria‐protective cytokines including granzyme B and IFN‐γ, but could be inhibited by the high expression of CTLA‐4 and TIM‐3 on slow responder T cells. CTLA‐4 competes with CD28, an NFκB activator,^132^ and upregulates the NFκB regulator IκBα while inhibiting the NFκB activator AKT.^133^ TIM‐3 specifically suppresses NFκB activation that results from TCR signaling.^134^ Secondly, CTLA‐4 can also reduce T cell activation by capturing and degrading CD80/CD86 from the APC surface,^135^ decreasing APC capacity to induce antigen‐specific immunity and inducing T cell anergy and tolerance. Thirdly, CTLA‐4 binding to DCs promotes the immunosuppressive indoleanime 2,3‐dioxygenase (IDO) pathway^136^, ^137^ which has been observed to result in Treg induction in CHMI.^74^

Since both CTLA‐4 and TIM‐3 are highly expressed on Foxp3^+^ cells,^138^, ^139^ *slow responders’* high expression of these ligands may signify higher baseline numbers of Tregs. In CHMI, subjects with significantly higher baseline transcript levels of Treg‐associated markers *CD27*, *IL2Ra*, *CD4*, *ICOS*, *IRF4*, and *Runx1* 140, 141, 142, 143 are worse at controlling parasitemia,^84^ and Treg induction in CHMI results in impaired central memory development.^69^ TGF‐β, which is required to drive Treg differentiation,^144^ is one of the principal cytokine phenotypes observed in CHMI.^60^

Beyond inducing Tregs, TGF‐β production may be a regulatory mechanism in slow responders, as its production as a first‐wave response to CHMI abrogates expression of inflammatory protective cytokines.^60^ Beyond that, TGF‐β has a vast array of immunosuppressive functions including suppression of monocyte, DCs, B cell, and NK cell function (reviewed in Refs. 145, 146). Over the course of CPS immunization, serum TGF‐β levels in fast responders decrease or remain stable while serum TGF‐β levels in slow responders increase. TGF‐β may therefore drive an immunoregulatory feedback loop in slow responders: TGF‐β production leads to increased polarization of Tregs which express CTLA‐4 and TIM‐3, driving more TGF‐β production and resulting in elevated serum levels.

These pathways hypothetically bridge the gap between the high inhibitory ligand phenotype of slow responders and their reduced protective capacity after immunization, and are avenues for future investigation in CHMI.

## 6 FACTORS UNDERLYING IMMUNOLOGICAL HETEROGENEITY

In addition to being a powerful tool to characterize heterogeneity in immune responses to malaria both at the individual and population level, CHMI can also help to identify both external and intrinsic factors underlying such heterogeneity. Depending in part on whether a given factor is naturally prevalent in the respective subject population, CHMI design in malaria‐naive and/or endemic populations can take account of these factors at up to three levels: they can be measured and accounted for in a multiparameter analysis (eg for internal, immutable factors); they can be selected for as a condition of (stratified) inclusion; or they can in some cases even be experimentally manipulated (randomly or otherwise).

### 6.1 External factors

At a global level, malaria vaccine efficacy has paradoxically often been found to be higher in malaria‐naive volunteers than in endemic populations. Two main extrinsic factors have been proposed to explain this marked difference: immunomodulation due to prior exposure to malaria^13^, ^16^ and due to co‐endemic infections.^17^, ^18^

As previously noted, precise determination of the quality, quantity and timing of an individual's prior exposure to *Plasmodium* parasites in the field is difficult. Nevertheless, a crude estimate thereof may be inferred from serological responses to panels of parasite antigens^91^, ^147^ and used purposefully to stratify participants in CHMIs in endemic populations.^48^, ^70^ More complex CHMI study designs, involving repeat infections, can also be employed to assess the effects of prior malaria exposure experimentally, either in endemic (PACTR ID: 201901672024347) or naive populations.

Antigenic and fitness diversity among parasite strains likely also represents an important factor explaining the relatively lower efficacy of candidate malaria vaccines under conditions of natural exposure.^82^, ^148^, ^149^ Indeed, developing broad strain‐transcending immunity is a major hurdle for malaria vaccines, as was highlighted by recent data indicating that low efficacy of the RTS,S/AS01 vaccine (Mosquirix^®^) in the field was partially due to epitope mismatch of CSP with circulating strains.^150^ The expanding portfolio of *Plasmodium* strains characterized and available for CHMI^37^, ^82^, ^151^, ^152^ allows assessment of parasite diversity as a factor both underlying heterogeneity in host immune responses and determining the level to which immunization strategies induce heterologous protection.^99^, ^148^, ^153^, ^154^

Tropical infectious diseases, in particular helminths, have been proposed as interfering conditional factors due to their immunomodulatory potential and overlapping geographical range.^18^, ^155^ Infection status can be identified at baseline using standard microbiological assays and used to stratify CHMI participants, or the effects of such infections on immune responses and protection in CHMI studies can be assessed by curatively treating (a subset of) participants prior to challenge. In theory, the effect of helminth co‐infections on malarial immunology could also be assessed experimentally using hybrid CHMI designs involving concomitant controlled infections with eg schistosomiasis or hookworm.^156^, ^157^

More cosmopolitan pathogens are known to have similar impacts on host immunity, among them cytomegalovirus (CMV). CMV is suggested to alter the makeup of host immunity by causing massive expansion of CMV‐specific effector memory T cells.^158^, ^159^ Furthermore, CMV‐mediated immunomodulation is known to impact the clinical course of various bacterial and viral pathogens^160^ and could play a similar role in malaria. CMV inhibits lymphoproliferative potential directly and through downregulation of DC function,^161^ which could lead to T cell inhibition. CMV also induces populations of NK cells with an “adaptive” phenotype, suggesting a role for *trained innate immunity*.^162^, ^163^ This recent new concept in immunology^164^, ^165^, ^166^ describes how particular microbial antigens, including bacille Calmette‐Guerin (BCG) vaccination^167^, ^168^, ^169^ and lipopolysaccharide,^170^ but also *Plasmodium spp.*,^171^, ^172^, ^173^ can induce epigenetic modifications in innate cells which alter responsiveness to heterologous stimuli. Indeed, subjects receiving BCG five weeks prior to malaria challenge develop higher levels of circulating IFN‐γ in response to CHMI,^174^ leading to higher NK cell cytotoxicity that correlates with delayed time to parasitemia. As such, modulation of the immune system due to prior exposure to common infectious, commensal or environmental microorganisms may be a major contributor to heterogeneity in CHMI.

### 6.2 Intrinsic factors

Even among endemic populations, malaria vaccine efficacy can appear higher in adults^175^ than in children and infants.^4^ It has been proposed that intrinsic age‐dependent maturation of the immune system^176^, ^177^, ^178^, ^179^ may underlie this. The importance of host age is more difficult to assess in the context of CHMI studies, since the ethics of doing challenge studies in children and infants is the topic of ongoing debate. However, this question may yet be addressed in the framework of CPS studies, which are both safer than standard CHMI and aim to induce protective immunity, thus satisfying the ethical criterion of direct benefit to the participant. Finally, DNA sequencing of CHMI participants can be performed to seek genetic explanations for heterogeneity in their immune response to malaria parasites. Nevertheless, studying monozygotic and dizygotic twins reveals that up to 58% of immune heterogeneity between individuals is non‐heritable.^180^ We therefore believe that it is unlikely that fast and slow responder phenotypes will prove to have a predominantly genetic basis.

Ultimately many factors, an interplay of genetic makeup and environmental exposure, must contribute to heterogeneity at individual and population level. Rare factors, factors that exert only a minor effect and/or factors that are unamenable to experimental manipulation, will be difficult to identify in the context of CHMI studies, which generally include only small numbers of subjects. Indeed, some factors influencing the immune response to malaria may never be resolved. Nevertheless, many such factors will likely act through final common immunological pathways. We have identified some such pathways in the context of CHMI studies, and others will follow. Further elucidation thereof may suggest potential interventions, eg specific adjuvants, to overcome sub‐optimal responses to malaria (vaccines) in individuals and populations, whatever the underlying cause(s).

## 7 CONCLUDING REMARKS: ELUCIDATING HETEROGENEITY TO DEFINE PATHWAYS OF PROTECTIVE IMMUNITY

**Figure 5:**
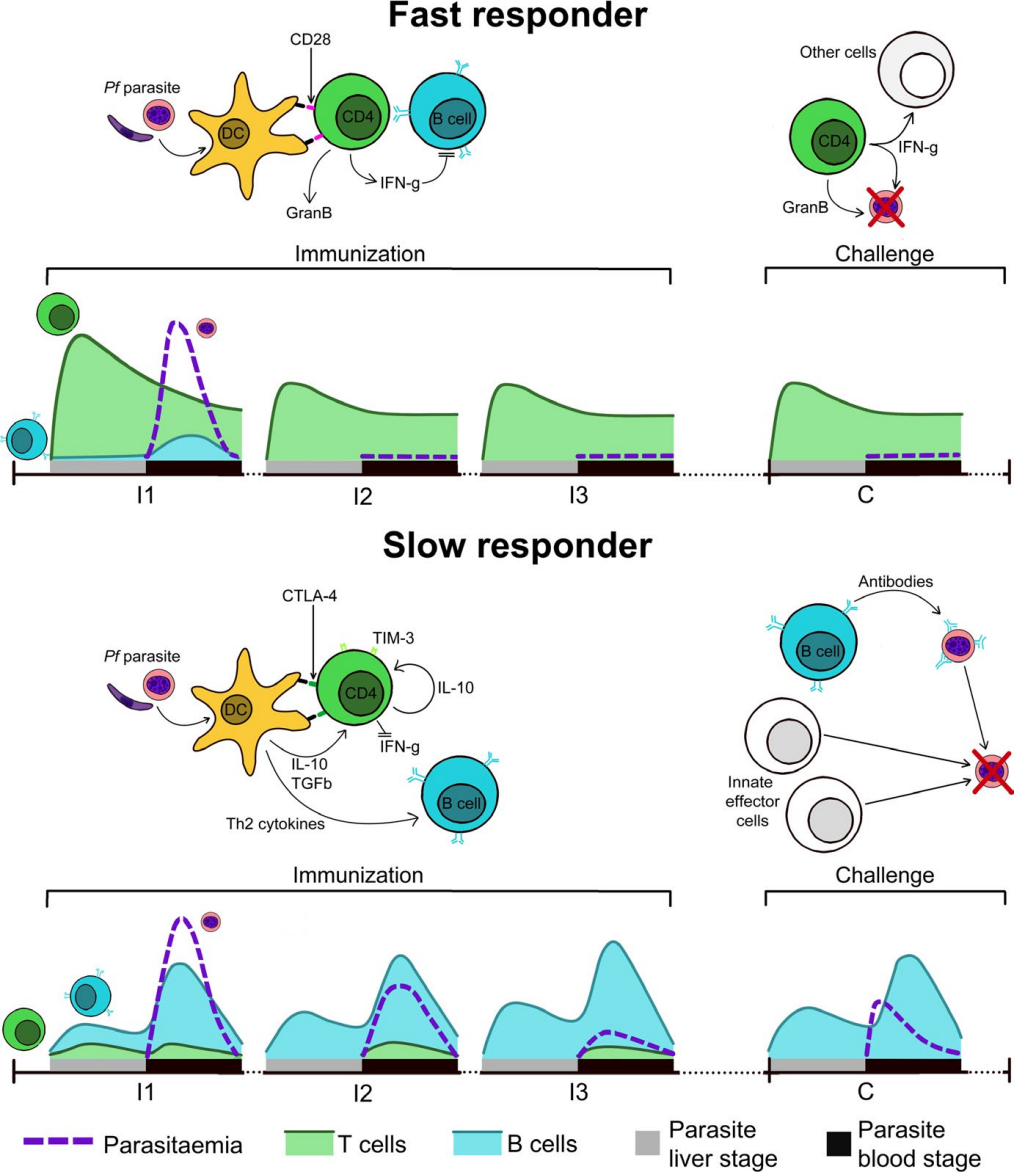
Fast and slow responders. Chemoprophylaxis with sporozoite immunization volunteers are exposed to three immunizations (I1‐I3) at monthly intervals followed by a challenge period (C) 2‐3 mo later. Based on the speed at which they acquire sterile protection, volunteers can be divided into fast and slow responders. Fast responders rapidly initiate cytotoxic and IFN‐γ^+^ T cell responses to malaria, but humoral responses are minimal, possibly due to inhibition by IFN‐γ^+^. Fast responders are successfully able to control parasitemia after the first immunization and are protected during final challenge by this stably maintained population of cytotoxic T cells. Slow responders do not initiate T cell responses to parasitemia as their T cells express high levels of inhibitory ligands CTLA‐4 and TIM‐3, leading to suppression of IFN‐γ in part due to Treg polarization. Instead, they produce humoral responses in response to the high burden of blood‐stage parasites. These antibodies are able to reduce parasitemia in subsequent immunizations but not control it entirely. Increased parasite exposure boosts antibody titers. During challenge, parasitemia is controlled through a mixture of humoral and innate responses, though this is less effective than IFN‐γ^+^ T cell immunity

The first CHMIs were given as a cure for syphilis. Increasingly, they may be a key to the very disease they inflict by unravelling the unknowns of antimalarial immunity. By studying immune responses after single CHMIs and CPS immunization regimens we found that volunteers are generally predisposed, by the inhibitory ligand expression of their CD4^+^ T cells, to respond to their first malaria encounter in a fast or slow fashion (Figure 5). Fast responders usually have an inflammatory phenotype, rapidly initiating T cell responses that promote IFN‐γ production and effectively control parasitemia through CD107a^+^ and granzyme B^+^ T cells but may suppress humoral immunity. Slow responders are instead generally predisposed to a tempered phenotype and suppress their IFN‐γ^+^ T cell responses, but compensate through the development of other mechanisms including humoral immunity that may eventually lead to their being protected. Some slow responders may never reach the immune threshold required for protection.

This suggests that heterogeneity in human immunity may be closer to a binary than a continuum. By drawing out these trends we hope to establish a new way of understanding immunity to malaria, and what should be considered correlate(s) of protection in this context. Examining this phenomenon more closely in field settings to elucidate its impact upon vaccination will provide some crucial insight into why some vaccines fail. Individual predisposition towards specific immune responses also has implications for future vaccine development: a one‐size‐fits‐all approach may never provide 100% protection across a whole population, since vaccines aiming to induce cellular responses in a slow responder may not have the expected protective efficacy, or vice versa. Identifying fast and slow responders prior to immunization could improve vaccine success, eg by administration of separate vaccines to fast and slow responders. Alternatively, adjuvant strategies may be able to compensate for individuals’ immune predilections, by modifying an “incompatible” phenotype to one which synergizes with that particular vaccine's mechanism of action. Such adjuvant strategies may still need to be tailored to an individual's immunological predisposition, as for example administration of an inflammatory adjuvant in someone with an already inflammatory phenotype might result in unacceptable immunopathology.

We have hypothesized as to the roles of a variety of factors in determining one's role as a slow or fast responder, but the mutability of this phenotype is an open question. There is some room for complexity beyond the binary: some fast responders are unprotected, and in the fully protected, induction of T cells is only a starting point in an immunological cascade that culminates in protection. What kind of stimulus might be able to change a slow responder to a fast responder? To what extent can external factors such as co‐infection affect an individual's ability to become a fast responder to malaria at any given time? Indeed, understanding the fundamental extent to which an individual's immune tendencies can be manipulated has vital implications for vaccine and adjuvant design. Conversely, to what extent can CPS immunization or malaria affect an individual's responsiveness to other pathogens? Could CPS immunization provide beneficial non‐specific effects similar to those seen with BCG, turning an individual into a fast responder to entirely different pathogens? Future research, and future CHMIs, should be designed with the aim of understanding how an individual becomes a slow responder, and how (if at all) they can be converted to a fast responder. Tackling heterogeneity before the first jab may universally increase vaccine success.

## CONFLICT OF INTEREST

The authors declare that this research was conducted in the absence of financial or commercial relationships that could construe a conflict of interest.
